# Quantification of Regulatory T Cells in Septic Patients by Real-Time PCR–Based Methylation Assay and Flow Cytometry

**DOI:** 10.1371/journal.pone.0049962

**Published:** 2012-11-27

**Authors:** Roman Tatura, Michael Zeschnigk, Michael Adamzik, Michael Probst-Kepper, Jan Buer, Jan Kehrmann

**Affiliations:** 1 Institute of Medical Microbiology, University Hospital Essen, University of Duisburg-Essen, Essen, Germany; 2 Institute for Human Genetics, University Hospital Essen, Essen, Germany; 3 Department for Anaesthesiology and Intensive Care Medicine, University Hospital Essen, Essen, Germany; 4 Institute for Clinical Transfusion Medicine, Städtisches Klinikum Braunschweig gGmbH, Braunschweig, Germany; Charité, Campus Benjamin Franklin, Germany

## Abstract

During sepsis, a relative increase of regulatory T (Treg) cells has been reported. Its persistence is associated with lymphocyte anergy, immunoparalysis and a poor prognosis. Currently, an exact quantification of human Treg cells based on protein expression of marker molecules is ambiguous, as these molecules are expressed also by activated non-regulatory T cells. Furthermore, no firm criteria for flow cytometer gate settings exist so far. Recently, a specific DNA methylation pattern within *FOXP3-TSDR* has been reported that allows distinguishing Treg and non-regulatory T cells, independent of their activation status. Using this epigenetic marker, we established a single-tube real-time PCR based methylation assay (QAMA) for relative quantification of Treg cells. Validation was performed on defined ratios of methylated and unmethylated target sequence and on mixtures of Treg and non-regulatory T cells. DNA-methylation was measured in CD4^+^ T cells isolated from blood samples of 30 septic patients and 30 healthy subjects and compared with results of Treg cell quantification by flow cytometry based on CD4^+^ CD25^hi^CD127^low^ measurement. In septic patients both methods showed an increased ratio of Treg cells to all CD4^+^ T cells. In healthy individuals, the results obtained by both methods were clearly positively correlated. However, the correlation between both methods in septic patients was only weak. We showed that quantification of Treg cells by QAMA detects CD4^+^ T cells with unmethylated *FOXP3-TSDR*, hidden in the CD25^med/low^ fraction of flow cytometry. Given that unmethylated *FOXP3-TSDR* is the most specific feature of Treg cells to date, our assay precisely quantifies Treg cells, as it additionally detects those committed Treg cells, hidden in the CD25^med/low^ fraction of CD4^+^ cells. Furthermore, QAMA is a reliable method, which is easier to standardize among laboratories and can thus improve reproducibility of Treg cell quantification.

## Introduction

Sepsis (systemic inflammatory response syndrome with infection) is still the leading cause of death in noncoronary European intensive care units [1]. In spite of medical progress and intensive research, the prognosis for septic patients has not essentially improved, and mortality rates for patients with severe sepsis still range from 30% to 50% [2]. The incidence of severe sepsis in Germany is 110 cases per 100,000, and approximately 30% of the budget for intensive care units (ICUs) is spent on treating septic patients [2].

During the past 30 years, essential progress has been achieved in understanding the pathophysiology and molecular mechanisms of sepsis. For years, mortality due to sepsis was believed to be caused by over-activation of the innate immune system and by the excessive proinflammatory response to severe microbial infection or tissue damage [3]. As anti-inflammatory therapies failed in clinical trials, it became obvious that sepsis could not be exclusively attributed to an uncontrolled proinflammatory response. Although some patients die during the state of hyper-inflammation, most deaths occur at later time points in the disease process. These later stages are associated with immunosuppressive conditions, characterized by the inability to clear the primary infection and a predisposition to secondary nosocomial infections [3]. It has been suggested that patients who survive sepsis but die later are those in whom the immune function does not recur [4] and that persistence of a pronounced immunoparalysis after septic shock is associated with a poor outcome [5].

Regulatory T (Treg) cells have been suggested to play a causative role in the progression and establishment of immune dysfunction in various diseases including sepsis [6]; [7]. The ratio of Treg cells, as quantified by the phenotypic protein expression analysis, has been reported to be elevated in septic mice and humans [6]–[9]. In this context, the persistence of an elevated ratio of Treg cells after the acute phase of sepsis has been associated with a poor long-term prognosis [10].

Treg cells are usually characterized by the expression of CD4, CD25, and Foxp3 and display a strong suppressive function on the proliferation of effector T cells. Foxp3, a transcription factor crucial to the development and function of Treg cells, has been assumed to be specific for Treg cells in mice and humans. Recently, however, it became obvious that activated human non-regulatory T cells also express Foxp3 but do not exhibit suppressive function [11]; [12]. So far, because of the lack of a specific Treg cell marker protein, it has not been feasible to distinguish between human Treg cells and activated T cells at the protein or mRNA level [13]. Recently, epigenetic differences in DNA methylation within *FOXP3* have been detected between Treg cells and non-regulatory T cells [14], which offer a promising option for the exact quantification of natural Treg cells.

In this report we describe the establishment of a quantitative analysis of methylated alleles (QAMA) assay that is based on epigenetic differences within the *FOXP3 Treg-specific demethylated region* (*TSDR*) between Treg cells and all other major blood cells. This assay allows the relative quantification of Treg cells in a single reaction tube. After validation on defined ratios of methylated and unmethylated target sequence as well as on mixtures of Treg and non-regulatory T cells, we used QAMA for quantification of Treg cells in blood samples from septic patients, which is a collective of patients with a high degree of activated immune cells, and healthy subjects and compared the results with those obtained by flow cytometric analysis for quantifying Treg cells (CD4^+^ CD25^hi^CD127^low^).

**Figure 1:**
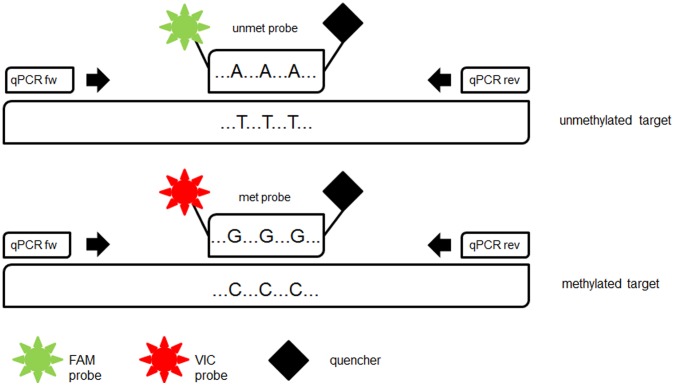
Schematic illustration of *FOXP3* Treg-specific demethylated region (*TSDR*) quantitative analysis of methylated alleles (QAMA) assay. *FOXP3-TSDR*-QAMA is a single tube quantitative real-time PCR. Bisulfite-treated target sequence is amplified with a single primer set irrespective of its methylation status. Two different labelled internal MGB Taqman® probes bind specifically to the methylated or unmethylated target sequence and are cleaved by the 5′nuclease activity of *Taq* DNA polymerase. The amount of fluorescence dyes VIC and FAM released during PCR is directly proportional to the amount of PCR product generated from the methylated or unmethylated allele. FAM probe = 6-carboxyfluorescein met probe = probe specific for methylated target unmet probe = probe specific for unmethylated target VIC probe = 4,7,2′-trichloro-7′-phenyl-6-carboxyfluorescein.

**Figure 2:**
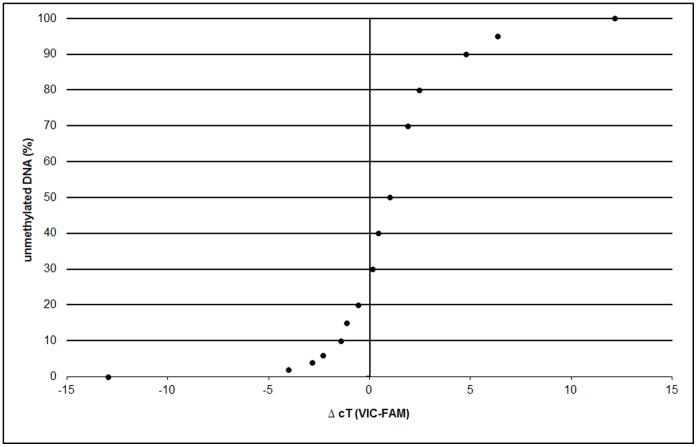
*FOXP3-TSDR* standard curve. The difference of both cT values (cT methylated probe – cT unmethylated probe) is determined and the methylation ratio of each sample deduced from a standard curve running along with each assay. ΔcT = difference in cycle-threshold values.

## Materials and Methods

### Ethics Statement and Study Population

Blood sampling from septic patients from the ICU of the Department for Anaesthesiology and Intensive Care Medicine of the University Hospital Essen was approved by the Ethics Committee at the University Hospital of Essen (North Rhine-Westphalia, Germany, no.: 06-3078). Written informed consent was obtained from all patients or from patient guardians, as appropriate. A total of 30 randomly chosen patients with severe sepsis or septic shock and 30 healthy subjects were enrolled in the study between January 2011 and April 2012. The diagnosis was based on the criteria of the American College of Chest Physicians/Society of Critical Care Medicine (ACCP/SCCM) [15]. Blood samples were obtained from patients at least 48 hours after the onset of sepsis.

### Isolation of CD4^+^ T Cells from Blood

Blood samples (4 mL) were processed within two hours. CD4^+^ cells were isolated with the Dynal CD4 Positive Isolation Kit (Invitrogen, now Life Technologies, Darmstadt, Germany) according to the manufacturer’s guidelines. The CD4^+^ cells were at least 98% pure, as confirmed by flow cytometry. One part of the purified CD4^+^ cells was used for flow cytometric quantification of Treg cells; the other part was subjected to DNA extraction.

### Flow Cytometry

For cell surface immunostaining, we used fluorescein isothiocyanate (FITC)-, allophycocyanin (APC)-, and Pacific Blue (PB)-conjugated monoclonal antibodies against CD4 (clone RPA-T4), CD25 (clone BC96), and CD127 (clone eBioRDR5) (all from eBioscience, San Diego,CA).

**Table 1:** Characteristics of the septic patients enrolled in the study.

Category	Subcategory	Result (%)
Age	<25 y	2 (7)
	25–60 y	20 (66)
	>60 y	8 (27)
Sex	Male	14 (47)
	Female	16 (53)
SAPS II (at admission)	<30	3 (10)
	30–60	18 (60)
	>60	9 (30)
Catecholamines		30 (100)
Mechanical ventilation		29 (97)
Extra-renal support		22 (73)
Site of infection	Lung	19 (63)
	Abdomen	7 (23)
	Other	4 (13)
Type of infection	Ambulant	8 (27)
	Nosocomial	22 (73)
Documented cause of sepsis	Gram-negative bacilli	15 (50)
	Gram-positive cocci	11 (37)
	Fungi	4 (13)
Mortality rate		10 (33)

SAPS = Simplified Acute Physiology Score.

**Table 2:** Characteristics of healthy subjects enrolled in the study.

Category	Subcategory	Result (%)
Age	<25 y	1 (3)
	25–60 y	29 (97)
	>60 y	0 (0)
Sex	Male	15 (50)
	Female	15 (50)

**Figure 3:**
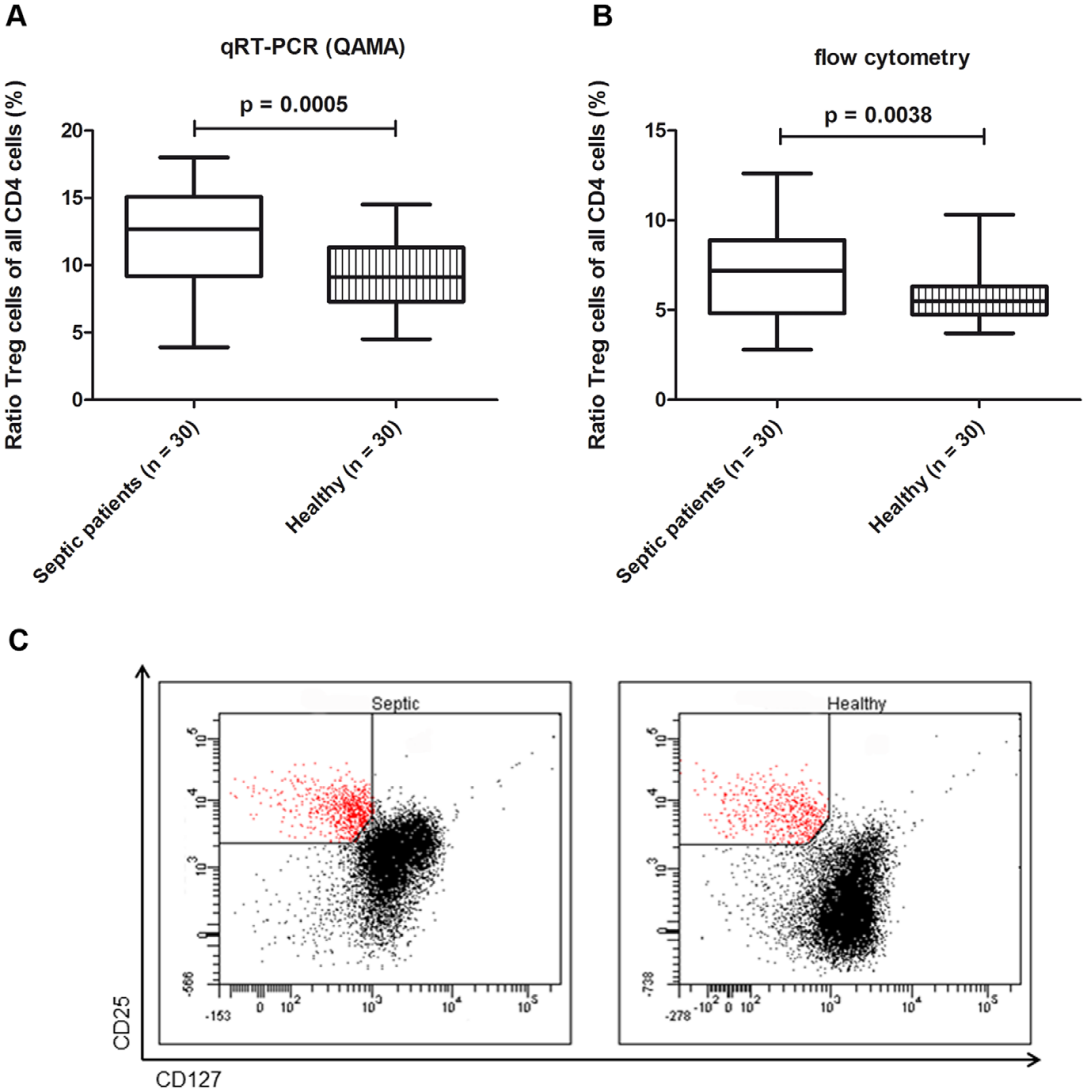
Quantification of Treg cells in septic patients and healthy individuals. A Relative quantification of Treg cells by *FOXP3* Treg-specific demethylated region (*TSDR*) real-time quantitative polymerase chain reaction (quantitative analysis of methylated alleles, QAMA) **B** Relative quantification of Treg cells (characterized as CD4^+^ CD25^hi^CD127^low^) by flow cytometry **C** Representative quantification of one septic patient and healthy subject by flow cytometry. Setting of the CD25 and CD127 flow cytometer gates are shown.

The mean ratio of Treg cells was significantly higher in septic patients (12.14%) than in healthy subjects (9.25%), as measured by *FOXP3-TSDR* QAMA (p = 0.0005). In septic patients, the ratio ranged from 3.9% to 18.0% and was broader than in healthy subjects (4.5% to 14.5%) (Figure 3a).

### DNA Extraction and Bisulfite Treatment

The isolation of DNA from blood was performed with the QIAamp DNA Mini Kit (Qiagen, Hilden, Germany) according to the manufacturer’s guidelines. Bisulfite modification of DNA was performed with an established protocol with minor modifications [16]. The DNA was recovered with the Wizard DNA Clean-Up System (Promega, Mannheim, Germany).

### Real-time Polymerase Chain Reaction (PCR)

PCR was performed in 96-well optical trays with a Roche LightCycler 480 system (Roche Diagnostics, Basel, Switzerland) to a final reaction volume of 20 µL, containing 10 µL 2-fold Roche TaqMan Probe Master 480, 2 µL bisulfite-treated DNA, 1 µM of each primer (FOXP3qPCRfw, GAAATTTGTGGGGTGGGGTATTTGTTTT; FOXP3qPCRrev, ATCTACATCTAAACCCTATTATCACAACCCCC). The probes (VIC-labeled methylated FOXP3, TCGGCGTATTCGG; FAM-labeled unmethylated FOXP3, AGTTTGGTGTATTTGGT) were added to a final concentration of 166 nM. All samples were analyzed in duplicate. After initial denaturation at 95°C for 10 min, the samples were subjected to 40 cycles at 95°C for 15 sec and at 60°C for 1 min. Primers were designed with the MethPrimer (LiLab, UCSF,CA, http://www.urogene.org) software, probes were designed with Primer Express software V2.0 (ABI, Carlsbad, CA).

### Standard Curve

Various ratios of methylated and unmethylated *FOXP3* template DNA were used to generate a standard curve. The *FOXP3* target region was amplified from a bacterial artificial chromosome (BAC; FOXP3, RPCIB753C201037Q, Imagenes, Berlin, Germany) in a reaction volume of 25 µL containing 5 µL of GoTaq reaction buffer and 0.125 µL of GoTaq polymerase (Promega, Fitchburg, WI) and 0,5 µM each of the primers FOXP3SeqTfw (TTCAGAGCTAGGGGCTTGTC) and FOXP3SeqTrev (GGACACTTGGCCAGAGCTAA). The deoxyribonucleotide triphosphates (dNTPs) were adjusted to a final concentration of 200 µM, and MgCl_2_ was adjusted to a final concentration of 1.5 mM. After activation at 94°C for 10 min, amplification was performed as follows: 40 cycles at 94°C for 45 sec, at 55°C for 45 sec, and at 72°C for 1 min.

PCR products were purified with the QIAquick Gel Extraction Kit (Qiagen, Hilden, Germany). The concentration of isolated DNA was determined with a NanoDrop ND-1000 spectrophotometer (peqLAB, Erlangen,Germany).

A fraction of the *FOXP3*-PCR product was methylated with CpG methyltransferase (*M. SssI*) (New England Biolabs, Ipswich, MA). Mixtures of 15 differing ratios of methylated and unmethylated DNA were prepared for the standard curve. DNA mixtures were treated with bisulfite-solution as described above.

### Statistical Analysis

For female samples, the percentage of Treg cells was corrected by a factor of two because one of the two *FOXP3-TSDR* alleles is methylated as a result of × inactivation. Pearson’s correlation coefficient and *t* test statistics were used to determine correlation. All reported *P* values were obtained with two-sided tests. Statistical analysis was performed with GraphPadPrism 5.0 software (Graph Pad Software, La Jolla, CA).

**Figure 4:**
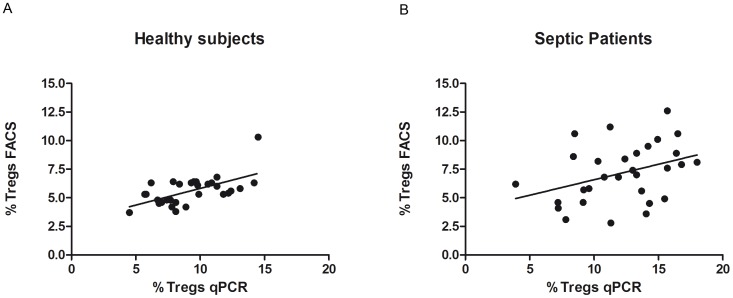
Correlation of qRT-PCR and flow cytometry. Correlation of ratio of Treg cells as quantified by *FOXP3-TSDR* QAMA qRT-PCR and as quantified by flow cytometry shows a clear positive correlation in healthy subjects (*r* = 0.60) and a weak positive correlation in septic patients (*r* = 0.37) FACS = fluorescence-activated cell sorting qRT-PCR = quantitative real-time polymerase chain reaction.

**Figure 5:**
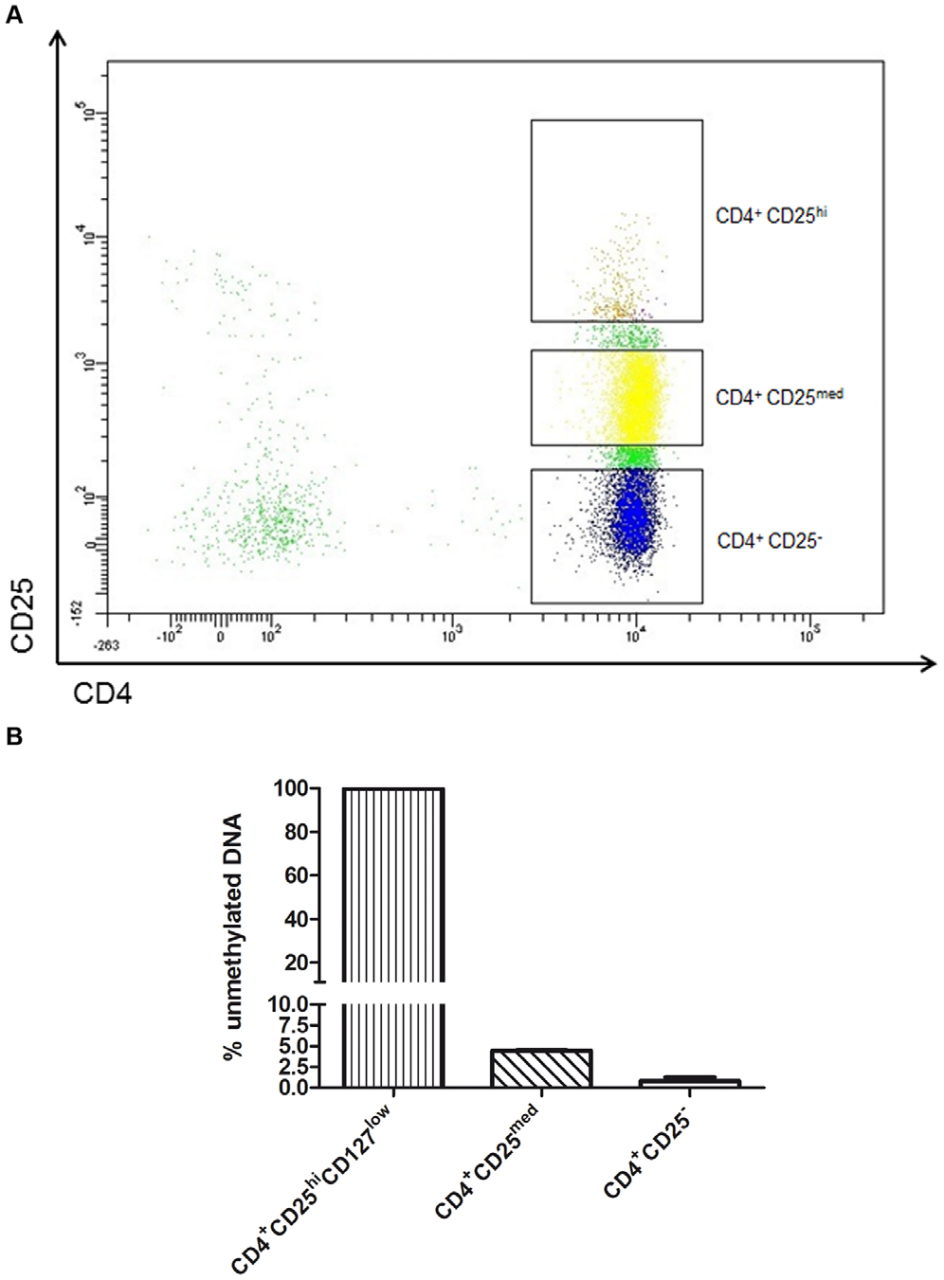
Illustration of the CD25 and CD4 gate settings used to sort various CD4^+^ populations. CD4^+^ CD25^med^ cells showed an unmethylated ratio of 4.4% (SD ±0.17%); CD4^+^ CD25^−^ cells, of 0.8% (SD ±0.60%); and CD4^+^ CD25^hi^CD127^low^ cells of 99.8% (SD ±0.24%)with *FOXP3-TSDR* QAMA. Results were calculated of four experiments from four healthy men.

## Results

### Establishment of a Methylation-sensitive Real-time *FOXP3-TSDR* Assay

For quantification of Treg cells by methylation-sensitive real-time PCR, we established a QAMA assay for *FOXP3-TSDR*. The QAMA method permits relative quantification of methylated and unmethylated DNA in the same reaction tube with only a single primer pair and the use of two Taqman MGB probes [17] (Figure 1). The differently labelled probes were designed to bind specifically to either the methylated or the unmethylated *FOXP3-TSDR* target sequence. To achieve maximum specificity, each probe covers three CpGs within the *TSDR*. For precise quantification of the ratio of methylated to unmethylated target sequence, the difference between the cycle-threshold values (ΔcT values) of both probes is determined. The methylation ratio is calculated with the help of a standard curve (Figure 2). The results obtained by the QAMA assay are largely independent of the total amount of DNA included in the assay over a broad range. To the best of our knowledge, there is no other method available for unbiased and precise Treg cell quantification that could be used for validation of the methylation based assay. Therefore, we analyzed various mixtures of methylated and unmethylated target DNA. In addition, we mixed various defined amounts of cells of a Treg cell line (TregTHU [18]) with cells from a non-regulatory T-cell line (CD4-39 [18]) and of FACS sorted human Treg (unmethylated) with non-regulatory T cells (methylated) and precisely quantified each mixture by using the *FOXP3-TSDR* QAMA assay (see Table S1 and Table S2).

### Quantification of Natural Treg Cells in Septic Patients and Healthy Subjects by Methylation-sensitive Real-time *FOXP3-TSDR* PCR

Blood samples from 30 septic patients and 30 healthy subjects were obtained between January 2011 and April 2012. The characteristics of the septic patients and healthy subjects are shown in Table 1 and Table 2.

### Quantification of Natural Treg Cells in Septic Patients and Healthy Subjects by Flow Cytometry (CD4^+^ CD25^hi^CD127^low^)

Treg cells quantified by flow cytometry were characterized by the expression of CD4^+^ CD25^hi^CD127^low^. We found that the mean ratio of Treg cells to all CD4^+^ T cells was significantly higher in septic patients (7.16%) than in healthy subjects (5.6%; p = 0.0038). This ratio ranged from 2.8% to 12.6% in septic patients and from 3.7% to 10.3% in healthy subjects (Figure 3b).

### Correlation of Quantification of Treg Cells as Determined by Flow Cytometry and by Methylation-sensitive Real-time PCR

For healthy subjects, there was a clear positive correlation (r = 0.60) between the results obtained with the *TSDR* QAMA assay and those obtained with flow cytometry (Figure 4A). In contrast, this correlation was weak (r = 0.37) in septic patients (Figure 4B).

For 29 of 30 healthy subjects and for 27 of 30 septic patients the ratio of Treg cells to all CD4^+^ cells as determined by *TSDR* QAMA assay was higher than that as determined by flow cytometry. To assess the reason for this discrepancy, we analysed if some Treg cells with unmethylated *FOXP3-TSDR* may be detected within the CD4^+^ CD25^med^ and CD4^+^ CD25^−^ populations.

### Methylation Analysis of Different CD25-expressing CD4^+^ T Cell Populations

Analysis of different populations of CD25-expressing CD4^+^ T cells of four healthy men showed that 99.8% of CD4^+^ CD25^hi^CD127^low^ cells are unmethylated within *FOXP3-TSDR*, while 4.4% of CD4^+^ CD25^med^ and 0.8% of CD4^+^ CD25^−^ T cells are unmethylated (Figure 5). QAMA captures unmethylated cells, hidden in the CD4^+^ CD25^med^ and CD4^+^ CD25^−^ population of flow cytometry.

## Discussion

This is the first study to analyze the relative ratio of Treg cells in blood of septic patients by utilizing the unique epigenetic signature of these cells within *FOXP3-TSDR*. Previous studies quantifying Treg cells in septic patients characterized these cells by phenotypic protein expression combining various molecules [6]–[8]; [19]–[21].

In recent years it has become obvious that it is currently unfeasible to obtain a precise relative quantification of Treg cells in humans by analyzing protein expression ex vivo without further manipulation [22]. The characteristic protein expression phenotype of Treg cells, including Foxp3, is also transiently exhibited by non-regulatory T cells, most notably in conventional CD4^+^ lymphocytes after activation [23]; [24]. Many studies have characterized Treg cells by using a combination of the interleukin (IL)-2 receptor alpha chain CD25 and the IL-7 receptor alpha chain CD127, but this combination also does not sharply discriminate Treg cells from conventional CD4^+^ T cells [25]. The ratio of Treg cells to the total number of CD4^+^ T cells depends essentially on the unstandardized placement of the CD25 gate of the flow cytometer within a smeared population of cells containing Treg cells and conventional CD4^+^ T cells and there is no firm criterion for the placement of the gate and the setting of the boundary within the CD25 positive population [25]. This drawback has so far hindered the reproducibility of Treg cell quantification in clinical settings, particularly under inflammatory conditions in which activated non-regulatory T cells express CD25 [25].

Another issue possibly impairing Treg cell quantification by flow cytometry is the recently published observation that murine natural Treg cells partially lose their characteristic Foxp3 expression but robustly reacquire Foxp3 expression and a strong suppressive activity upon stimulation of the T-cell receptor (TCR) [26]. These cells have been named “latent” Treg cells [26]. It has been previously assumed that Foxp3 is stably expressed in natural Treg cells. However, a similar loss of protein expression in human natural Treg cells would make their quantification even more difficult.

Epigenetic differences between Treg and non-regulatory T cells offer a diverse option for their quantification. Genomic DNA methylation is an important epigenetic modification that is generally stable over time in somatic differentiated cells [27]. The unmethylated *FOXP3-TSDR* is specifically existent only in natural/committed Treg cells in contrast to recently activated conventional T cells that transiently express Foxp3 or to other blood cells [14]. This epigenetic attribute provides an exclusive and the most specific marker for human natural Treg cells known so far [14]. Wieczorek and colleagues [13] established a real-time PCR analysis of *FOXP3-TSDR* for the quantification of human Treg cells. Our QAMA-methylation assay is beneficial over most other assays in two major aspects. It is largely independent of the total amount of DNA included and allows the relative quantification in a single reaction tube. This notably provides a high degree of robustness to the assay which is a prerequisite for an assay to be used in routine testing.

Using the *FOXP3-TSDR* QAMA assay, we found that the overall ratio of Treg cells to all CD4^+^ cells was significantly higher in septic patients than in healthy subjects. This finding is consistent with the results obtained by flow cytometry. Although septic patients and healthy subjects are not precisely matched for age, the different results cannot be explained by the different composition of the age of both groups in our analysis. We at least did not observe any significant difference in a subanalysis within the group of Treg cells between the analyzed eight septic patients >60 years of age and 22 patients <60 years of age (FACS in septic patients: >60 years, mean Treg cell ratio 5.7%; <60 years 7.4%; qRT-PCR: septic patients >60 years 12.6%; <60 years 12.0%). Omitting the results of the patients >60 years, there still is a significant increase of the Treg cells ratio in septic patients, compared to healthy subjects.

There are indeed reports of increased Treg cell ratio especially in mouse tissues correlated with age, but the picture is not clear in humans [28]. There are reports of an increased age-related Treg cell ratio in blood [29]–[30], but other studies did not observe an increased Treg cell ratio from human blood [31]–[32]. All these studies quantified Treg cells by flow cytometry.

The ratio of Treg cells to all other CD4^+^ cells, as measured by the *FOXP3-TSDR* QAMA assay, was higher in 56 of 60 samples than the ratio as determined by flow cytometry. One reason for this deviation is lack of specificity of marker proteins and the unstandardized placement of the flow cytometer gates. Another reason could be the existence of “latent” Treg cells that have partly lost their typical phenotypic expression. Both aspects are supported by the *FOXP3-TSDR* QAMA analysis of the CD25^med^ population in samples from healthy males, which showed a fraction of 4.4% (SD ±0.173%) unmethylated cells (Figure 5). Using the methylation assay, it is possible to catch the committed Treg cells with unmethylated *FOXP3-TSDR*, hidden in the CD25^med^/CD25^−^ fraction.

When the results obtained by QAMA and flow cytometry were compared for each sample, a clear positive correlation was obtained for healthy subjects (*r* = 0.605), which further supports the reliability of the methylation based assay. A similar correlation between methylation-sensitive real-time PCR and flow cytometry for quantification of Treg cells in blood has been reported by others (*r* = 0.49 [33]; *r* = 0.74 [34]). For septic patients, the positive correlation of the results obtained by both methods was only weak (*r* = 0.37) (Figure 4). As recent findings show that even Foxp3, which is regarded as most specific molecule for Treg cells, is also expressed in non-regulatory T cells and its expression is crucially dependent of the cellś activation status [11]; [12], the weak positive correlation of both assays found in the collective of septic patients does not surprise. The CD25 expression of activated T cells is regarded as an important reason for hindering the reproducibility of clinical data analysing the number of Treg cells by flow cytometry under inflammatory conditions [25]. To date, the characterization and quantification of Treg cells in mice and humans have been based almost exclusively on phenotypic protein expression because no more specific alternative existed. Especially for human sepsis, methods using epigenetic differences may be more exact in quantifying Treg cells. However, we cannot exclude that under particular conditions *FOXP3* methylation in Treg cells as well as in non-regulatory T cells might change thus impairing methylation based Treg cell quantification. However, it is commonly accepted that DNA methylation in differentiated cells is more robust than protein expression [27] and it has already been demonstrated, that human non-regulatory T cells conserve their methylation status after activation [14].

The persistence of an elevated ratio of Treg cells to all CD4^+^ T cells during sepsis has been reported to be associated with a prolonged immunosuppression after the acute phase of sepsis, a predisposition for secondary nosocomial infections and a poor prognosis [7], [10], [20]. Apart from a currently lacking critical threshold, the quantification of the Treg cell ratio as an early sepsis marker cannot be expected to be superior to the widely used biomarkers like IL-6, procalcitonin or C-reactive protein, because the elevated Treg cell ratio is not specific for sepsis but has been shown to be elevated for instance in certain cancer patients such as hepatocellular carcinoma [35] and breast cancer [36].

Quantification of the Treg cell ratio from blood seems less suitable as an early biomarker to diagnose sepsis but rather as a marker which might be useful for monitoring immune suppression after the acute phase of sepsis. It was demonstrated, that Treg cell ratio is elevated in mice surviving polymicrobial sepsis. These mice die, if a secondary bacterial infection is induced with *Legionella pneumophila,* but they have an improved survival, if the Treg cell ratio was reduced by glucocorticoid-induced tumor necrosis factor receptor antibody (DTA-1) before inducing the secondary infection with *Legionella pneumophila* [20].

It is still unclear, whether the exact ratio of Treg cells to a certain time point in the course of sepsis is rather beneficial to predict immunosuppression and prognosis than the patientś individual changes of this ratio. Although the mean ratio of Treg cells in our study is higher in septic patients compared to healthy subjects as quantified by flow cytometry and methylation sensitive qRT-PCR, this is not the case for each patient enrolled in the study. There are individuals with a contrary ratio of Treg cells, elevated in QAMA-PCR and average or reduced in flow cytometry just as vice versa. Particularly for these patients with contrary results, it would be of interest to confirm, if a persistence of an elevated Treg cell ratio during sepsis does more exactly predict immunosuppression and prognosis if quantified by methylation analysis compared to flow cytometry. To answer this question, larger longitudinal studies are needed for immune monitoring Treg cells during the course of sepsis by flow cytometry and methylation analysis.

The study is limited by the fact that sampling of septic patients was not standardized to one time point after the onset of sepsis, but was performed just at least 48 hours after the onset of sepsis. This might contribute to the poor positive correlation between flow cytometry and QAMA-PCR results due to the different lymphocyte activation status during the cause of disease with consequently changing protein expression.

In human sepsis, immunotherapy may provide an option for substantially improving outcome. In the murine system, decreasing the percentages of CD4^+^ CD25^+^ Foxp3^+^ Treg cells in septic mice by neutralizing either IL-10 or transforming growth factor (TGF)-β have been shown to improve their survival [37]. Several researchers have appreciated the impact and value of immune monitoring in septic patients, including the quantification of Treg cells [38].

The quantification of Treg cells may become a useful tool for predicting prognosis or monitoring the course of disease or the effectiveness of immune therapies in certain autoimmune diseases, such as inflammatory bowel diseases, systemic lupus erythematosus, and rheumatoid arthritis; these studies have also shown that the dimension of imbalance between Treg cells and conventional T cells is correlated with disease severity [22]. Additionally it could be useful in certain tumour diseases such as breast cancer, where the quantification of Treg cells has been used to determine which patients are at risk of late relapse [36].

Both methods, QAMA and flow cytometry showed an elevated number of Treg cells in septic patients. Given that unmethylated *FOXP3-TSDR* is the most specific feature of Treg cells to date, our assay precisely quantifies Treg cells, as it additionally detects those committed Treg cells, hidden in the CD25^med/low^ fraction of CD4^+^ cells. The unmethylated *FOXP3-TSDR* methylation pattern distinguishes Treg cells from activated non-regulatory T cells, which is advantageous especially under inflammatory conditions. *FOXP3-TSDR* QAMA is a reliable method, which is easier to standardize among laboratories and can thus improve reproducibility of the quantification of Treg cells, as there is no need to find firm criteria for setting a boundary within the fuzzy CD25 population containing Treg cells and non-regulatory T cells.

## Supporting Information

Table S1 **Quantification of various ratios from Treg cell mixtures.** Various Treg cell ratios are quantified from mixtures of sorted Treg cells and CD4^+^ CD25^−^ T cells and from a Treg cell line and a non-regulatory T cell line
(DOCX)

Table S2 **Quantification of various ratios from unmethylated and methylated** ***FOXP3*** **-DNA mixtures.**
(DOCX)
