# Supplementary material for: Quantification of Regulatory T Cells in Septic Patients by Real-Time PCR–Based Methylation Assay and Flow Cytometry
Source: PLoS One. 2012 Nov 27;7(11):e49962. doi: 10.1371/journal.pone.0049962 (PMC3507919; doi:10.1371/journal.pone.0049962)
Supplement: Table S1 — Quantification of various ratios from Treg cell mixtures. Various Treg cell ratios are quantified from mixtures of sorted Treg cells and CD4+ CD25− T cells and from a Treg cell line and a non-regulatory T cell line (DOCX) [file pone.0049962.s001.docx]

**Table S1. Quantification of various ratios from Treg cell mixtures**

|  | **Sorted T cells (Treg/Tconv**  **[CD4^+^CD25^hi^CD127^low^/CD4^+^CD25^-^])** | | **Cell line (Treg/Tconv) Mixture** |
| --- | --- | --- | --- |
| **Treg cell ratio (%)** | **Mixture 1 (%)** | **Mixture 2 (%)** | **Mixture (%)** |
| 20% | 19,40 | 19,54 | 20,41 |
| 10% | 9,63 | 10,04 | 9,66 |
| 5% | 5,88 | 5,29 | 6,28 |
| 2,5% | 2,59 | 2,81 | 2,46 |
| 1,25% | 1,30 | 1,39 | 1,43 |
