# Supplementary material for: Quantification of Regulatory T Cells in Septic Patients by Real-Time PCR–Based Methylation Assay and Flow Cytometry
Source: PLoS One. 2012 Nov 27;7(11):e49962. doi: 10.1371/journal.pone.0049962 (PMC3507919; doi:10.1371/journal.pone.0049962)
Supplement: Table S2 — Quantification of various ratios from unmethylated and methylated FOXP3 -DNA mixtures. (DOCX) [file pone.0049962.s002.docx]

**Table S2. Quantification of various ratios from unmethylated and methylated *FOXP3*-DNA mixtures**

|  | **methylated / unmethylated DNA mixtures from PCR product** | |
| --- | --- | --- |
| **unmethylated *FOXP3-TSDR* (%)** | **Mixture 1 (%)** | **Mixture 2 (%)** |
| 0% | 0,24 | -0,72 |
| 2% | 1,39 | 1,57 |
| 4% | 3,19 | 3,48 |
| 6% | 5,16 | 4,80 |
| 10% | 8,55 | 9,22 |
| 15% | 16,17 | 15,72 |
| 20% | 18,67 | 20,36 |
| 30% | 31,13 | 30,32 |
| 50% | 51,60 | 50,91 |
| 100% | 100,44 | 99,83 |
